# Biomaterials for the Treatment of Alzheimer’s Disease

**DOI:** 10.3389/fbioe.2016.00049

**Published:** 2016-06-16

**Authors:** Darya Hadavi, André A. Poot

**Affiliations:** ^1^Department of Biomaterials Science and Technology, Institute for Biomedical Technology and Technical Medicine (MIRA), University of Twente, Enschede, Netherlands

**Keywords:** Alzheimer’s disease, biomaterials, delivery systems, nanoparticles, liposomes, scaffolds

## Abstract

Alzheimer’s disease (AD) as a progressive and fatal neurodegenerative disease represents a huge unmet need for treatment. The low efficacy of current treatment methods is not only due to low drug potency but also due to the presence of various obstacles in the delivery routes. One of the main barriers is the blood–brain barrier. The increasing prevalence of AD and the low efficacy of current therapies have increased the amount of research on unraveling of disease pathways and development of treatment strategies. One of the interesting areas for the latter subject is biomaterials and their applications. This interest originates from the fact that biomaterials are very useful for the delivery of therapeutic agents, such as drugs, proteins, and/or cells, in order to treat diseases and regenerate tissues. Recently, manufacturing of nano-sized delivery systems has increased the efficacy and delivery potential of biomaterials. In this article, we review the latest developments with regard to the use of biomaterials for the treatment of AD, including nanoparticles and liposomes for delivery of therapeutic compounds and scaffolds for cell delivery strategies.

## Introduction

Neurological diseases, such as frontotemporal dementia, stroke-induced vascular dementia, and Alzheimer’s disease (AD), are characterized by the impairment of memory and cognitive functions (Han et al., 2015). More than 80% of dementia cases are caused by AD, which is categorized as one of the most occurring neurodegenerative diseases in the world (Anand et al., 2014). Moreover, AD is considered as a slow pandemic disease. Whereas currently, 3 out of 1000 individuals worldwide are suffering from AD, this is expected to increase to 12 per 1000 by the year 2050 (Brookmeyer et al., 2007).

Alzheimer’s disease can be classified as familial AD or sporadic AD, while they share clinical and pathological features, including progressive cognitive impairment, plaque deposits in the brain, axonal transport defects, synapse loss, and selective neuronal death. Only 5% of the AD cases are of familial type, whereas 95% belong to sporadic AD (Kim and Tsai, 2009; Thal and Fandrich, 2015). Another classification system for AD is based on the age of emerging. Whereas 1–6% of the AD cases emerges in 30- to 60-year-old people, which is known as early-onset AD, late-onset AD occurs in people older than 60 years with 90% prevalence (Mullane and Williams, 2013).

Alzheimer’s disease is characterized clinically by progressive decline in mental and cognitive ability. The disease begins with impairment in the part of the brain, which is responsible for learning, memorizing, thinking, and planning. This mental decline negatively affects the patients’ work and social life because they have difficulties in expressing themselves and organizing their thoughts (Alzheimer’s Association, 2012). As the disease progresses, more changes occur in the personality and behavior of the patients and they need extensive help with daily activities. Shrinkage of the brain over time results in more signs of AD, such as decline of thinking and reasoning skills, inability to respond to the environment or to control movement (Alzheimer’s Association, 2012).

Since Dr. Alois Alzheimer did observe the presence of amyloid plaques [extracellular deposits of amyloid-beta (Aβ) peptide] and intracellular hyperphosphorylated neurofibrillary tangles (NFTs) in the brain as pathological features, many hypotheses concerning causative factors for AD have emerged (Armstrong, 2013; Kumar et al., 2015). Three main causes of AD have been proposed. It can be due to dysregulation of the cholinergic system, which is related to cognitive impairment of the patient. Alternatively, AD can be due to accumulation of NFTs or deposition of Aβ peptide in the brain (Anand et al., 2014). NFTs are paired helical filaments resulting from hyperphosphorylation of tau proteins. These proteins are mostly found in the axons, and their hyperphosphorylation is caused by internal cell dysregulation. NFTs severely harm neurotransmitter transport and axonal integrity (Sun et al., 2003). The formation of Aβ peptides is the result of cleavage of amyloid precursor protein (APP). APP is a type I transmembrane protein, which is highly expressed in the central nervous system (CNS) and suggested to be responsible for synapse formation, neurogenesis, axonal transport, cell signaling, and plasticity. During two enzymatic steps by β-secretase and γ-secretase, Aβ peptides mainly consisting of 40 or 42 amino acids are liberated from APP, forming Aβ oligomers, Aβ polymers, and eventually Aβ plaques (Thinakaran and Koo, 2008). The presence of NFTs and Aβ plaques in the brain causes shrinkage pressure, which leads to nerve cell apoptosis. This cell death process starts from the memory and learning regions of the brain and then spreads to other regions *via* transfer among nerve cells that are along anatomical pathways (Gunawardena and Goldstein, 2001; Brunholz et al., 2012). Currently, the pathophysiology of AD is subject to changes. Recent studies claim that Aβ peptide and phosphorylated tau proteins are the reflection of damage in neurons rather than being responsible for the disease (Hardy and Selkoe, 2002). It has been suggested that formation of NFTs is due to an adaptive strategy of cells to oxidative stress over the long term. Nevertheless, among all hallmarks of AD, Aβ deposition is the prevailing one for more than two decades and considered as an important causative factor of AD (Hardy and Selkoe, 2002).

It has been shown that Aβ oligomers are the primary structures leading to damage of normal functions of the brain (Gu et al., 2009; Orive et al., 2009). Based on studies on familial AD, mutations were only seen in APP and presenilin genes (PS1 and PS2), and no mutations were reported for tau protein. This finding suggests that hyperphosphorylation and aggregation of tau protein in the form of NFTs result from the direct effect of Aβ peptides on tau protein (Ranka et al., 2002). The involvement of Aβ oligomers in synaptic impairment suggests that they have negative effects on postsynaptic structure and plasticity of the excitatory glutamatergic synapse (Shankar et al., 2008). As amyloid plaque formation is a rather late process (Orive et al., 2009), more insight in the formation of Aβ peptide is needed in order to get a comprehensive understanding of the etiology of this disease.

## Current Treatment

The enormous financial and emotional burden of AD on patients, community and caregivers as well as increasing numbers of AD patients raise the essential need for an effective treatment. About a century after the discovery of this disease, only five drugs have been approved by the US Food and Drug Administration (FDA): acetylcholinesterase inhibitors rivastigmine (Exelon), galantamine (Razadyne and Reminyl), tacrine (Cognex), donepezil (Aricept), and NMDA receptor antagonist memantine (Namenda) (Musardo et al., 2013). These drugs are only able to target symptoms of AD and decrease the speed of progression of the disease. However, many patients do not respond to these drugs or the drugs have no profound effects on alteration of the process of AD (Han et al., 2015).

From 1998 to 2011, approximately 100 therapeutic compounds were tested in clinical trials for the treatment of AD, but they all failed. Among others, agents included inhibitors of γ-secretase and Aβ oligomer/fibril formation, as well as antibodies against Aβ (Mullane and Williams, 2013). This raised doubt on the causality of Aβ in the development of AD (Armstrong, 2014). Moreover, it was suggested that patients were treated too late, having irreversible brain damage 10–20 years after onset of the disease, explaining the discrepancy with promising studies using animal models of AD (Mullane and Williams, 2013). Importantly, clinical failure of therapeutic agents was explained by low bioavailability in the CNS due to restriction by the blood–brain barrier (BBB) (Lauzon et al., 2015). In order to develop effective therapies for AD, much effort is needed to increase our knowledge of the involved disease pathways and to develop suitable drug delivery systems. With respect to the latter subject, the beneficial properties of biomaterials have been demonstrated, which are reviewed in this article for the treatment of AD.

As listed on the website http://clinicaltrials.gov, novel therapeutic compounds tested in clinical trials for the treatment of AD include the neurosteroid allopregnanolone (Irwin and Brinton, 2014), the mast cell inhibitor masitinib (Folch et al., 2015), and the glucagon-like peptide-1 mimetic Exendin-4 (Hölscher, 2014). These agents are able to cross the BBB and thus may be effective without a biomaterial delivery system. Also, insulin is being tested in several trials (Hölscher, 2014). To prevent systemic administration, the insulin is delivered intranasally, which may benefit from a drug delivery system as shown below. Moreover, administration of mesenchymal stem cells is entering clinical trials for the treatment of AD (Hunsberger et al., 2016), which may also benefit from delivery strategies using biomaterials as reviewed below.

## Biomaterials and Nanotechnology

As defined by Williams (2009), a biomaterial is “a material intended to interface with biological systems to evaluate, treat, augment, or replace any tissue, organ, or function of the body.” Biomaterials are widely used in the fields of drug delivery and tissue engineering. One of the increasing applications of biomaterials is to overcome the inherent inability of the brain to protect, repair, and regenerate itself (Orive et al., 2009). One of the major problems with respect to the treatment of neurodegenerative diseases is related to drug delivery. Drug availability in the CNS is low due to the BBB, which restricts the accessibility of therapeutic compounds to the CNS. The BBB is formed by tight junctions between endothelial cells lining the cerebral capillaries. Compared to other parts of the circulation, the tight junctions of the BBB are more extensive and pinocytosis by the endothelial cells is limited (Ballabh et al., 2004). The endothelial cells are surrounded by a basement membrane as well as pericytes and astrocytes, which provide structural and biochemical support to the BBB. The BBB protects the brain against pathogens, and only oxygen, carbon dioxide, and other lipophilic molecules with a molecular weight <600 g/mol can freely diffuse across the BBB. Other compounds, such as glucose, amino acids, and insulin, enter the brain *via* specific transporters or receptor-mediated endocytosis (Ballabh et al., 2004).

Because of the BBB, effective methods are needed for the delivery of therapeutic agents in the brain. Different approaches have been considered, such as conjugation of the active compounds to vectors, such as nanocarriers [liposomes, polymeric micelles, and lipid and polymeric nanoparticles (NPs)] with high affinity to the BBB, for instance, by interaction of the nanocarriers with various brain nutrient transport systems. An example of this is coating of NPs with thiamine, thereby targeting the particles to the BBB thiamine transporter (Lockman et al., 2003). Such vectors have the capacity of promoting active transport of drugs through the BBB (Popovic and Brundin, 2006; Beduneau et al., 2007). Moreover, implantation of drug-loaded biodegradable carriers, such as NPs and microparticles, enables delivery of a drug near the desired site of action. Recently, developed biomaterials can improve the targeted delivery of therapeutic compounds and cells to the brain, as reviewed below. These applications should lead to protection, repair, or regeneration of damaged CNS tissue (Orive et al., 2009).

**Figure 1:**
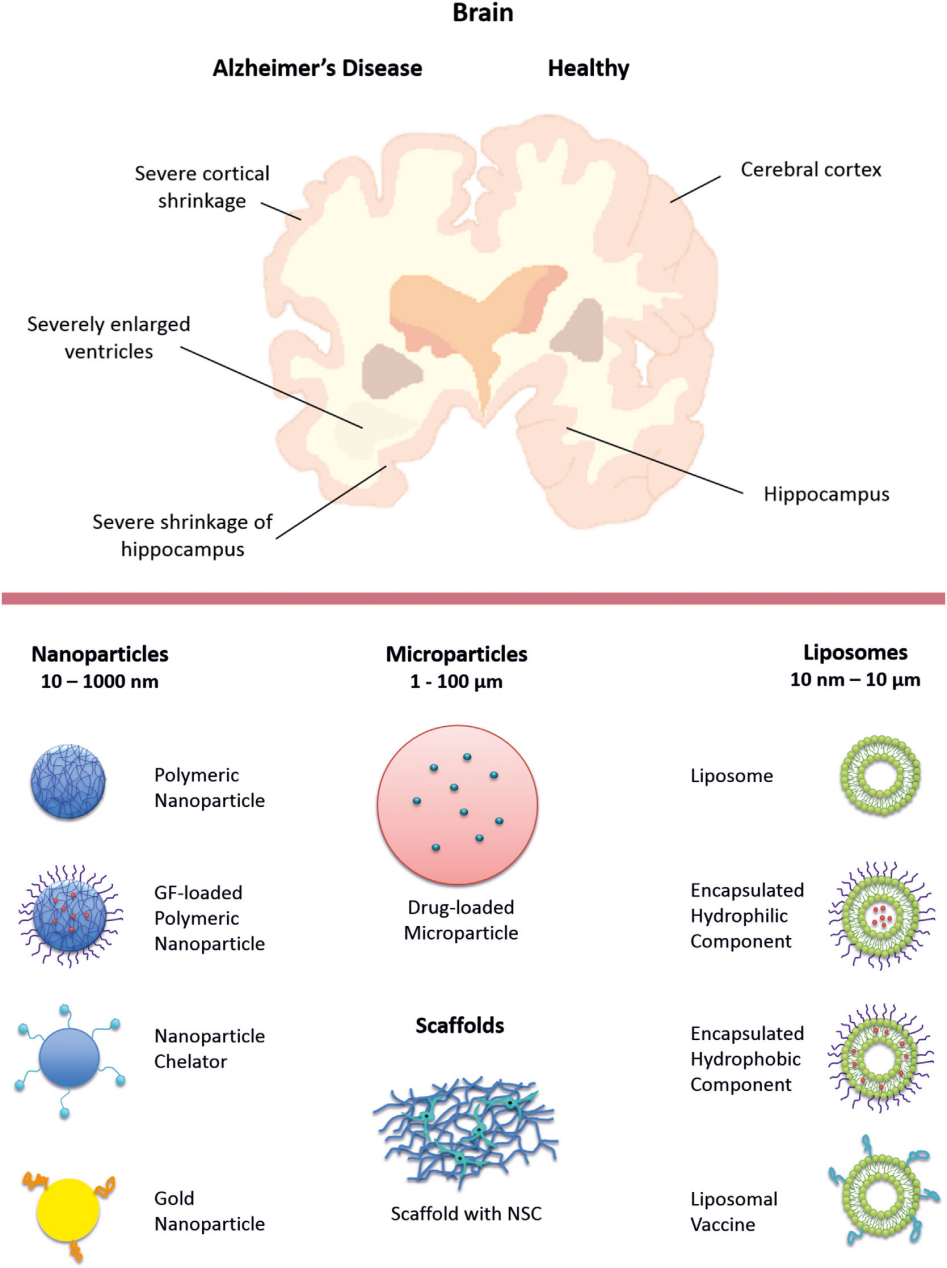
**Schematic representation of AD/healthy brain tissue (top) and the biomaterial delivery systems described in this article (bottom)**.

Nanotechnology is one of the most important fields in biotechnology and has a major role in the development of novel therapeutic modalities with increased efficacy. In the nanotechnology field, materials are engineered with functional organization on the nano scale (1–1000 nm). These materials are used for interaction with and stimulation of biological systems (e.g., cells or tissues) at the molecular level to induce physiological responses. Nanotechnology provides a route to get desired responses more selectively with better timing and lower side effects in comparison with conventional pharmacological approaches (Modi et al., 2009). Here, we review recent advances in the applications of nano-sized biomaterials for the treatment of AD, mainly based on the use of NPs and liposomal nanocarriers. The biomaterial delivery systems described in this article are schematically shown in Figure 1. Information on the accompanying therapeutic agents is listed in Table 1.

**Table 1:** **Therapeutic agents mentioned in this article: current status, way of administration, and therapeutic effect**.

Agent, section	Status	Administration	Effect
Rivastigmine, Current Treatment	FDA approved	Oral, plaster	Selective pseudo-irreversible inhibition of acetylcholinesterase
Galantamine, Current Treatment	FDA approved	Oral	Selective reversible inhibition of acetylcholinesterase
Tacrine, Current Treatment	FDA approved	Oral	Non-selective reversible inhibition of acetylcholinesterase
Donepezil, Current Treatment	FDA approved	Oral	Selective reversible inhibition of acetylcholinesterase
Memantine, Current Treatment	FDA approved	Oral	Non-competitive inhibition of the *N*-methyl-d-aspartate receptor
Allopregnanolone, Current Treatment	Clinical trial	Oral	Increased expression of brain LXR and PXR receptors
Masitinib, Current Treatment	Clinical trial	Oral	Inhibition of mast cells and tyrosine kinase fyn
Exendin-4, Current Treatment	Clinical trial	Oral	Mimetic of glucagon-like peptide-1, acts as neuronal growth factor
Insulin, Current Treatment	Clinical trial	Intranasal	Stimulation of cellular glucose uptake, growth factor-like properties
Mesenchymal stem cell, Current Treatment	Clinical trial	Intravenous or intracerebral injection of cell suspensions	Secretion of neurotrophic factors, differentiation to neuronal cells
IGF, Growth Factor-Loaded Polymeric Nanoparticles	Animal model	Intravenous or intranasal injection of NPs	Growth factor signaling
NGF, Growth Factor-Loaded Polymeric Nanoparticles	Animal model	Intravenous or intranasal injection of NPs	Growth factor signaling
bFGF, Growth Factor-Loaded Polymeric Nanoparticles	Animal model	Intravenous or intranasal injection of NPs	Growth factor signaling
Deferiprone, Nanoparticle Chelators	*In vitro* model	Addition of NPs	Inhibition or reverse of Aβ aggregation by chelation of metal ions
d-penicillamine, Nanoparticle Chelators	*In vitro* model	Addition of NPs	Inhibition or reverse of Aβ aggregation by chelation of metal ions
Clioquinol, Nanoparticle Chelators	Animal model, clinical trial	Oral	Inhibition or reverse of Aβ aggregation by chelation of metal ions
	Animal model	Intravenous injection of NPs	
Polyoxometalate/β-sheet breaker peptide, Gold Nanoparticles	*In vitro* model	Addition of gold NPs	Inhibition or reverse of Aβ aggregation by interfering with Aβ
Docosahexaenoic acid, Liposomes	*In vitro* model	Addition of liposomes	Induction of non-amyloidogenic processing of APP
Curcumin, Liposomal Encapsulation of Natural Components	*In vitro* model	Addition of liposomes	Targeting of liposomes to Aβ fibrils
Quercetin, Liposomal Encapsulation of Natural Components	Animal model	Oral, intranasal injection of liposomes	Reduction of oxidative stress
Aβ peptides, Liposomal Vaccines	Animal model	Intraperitoneal injection of liposomes	Immune response to Aβ peptides
Rivastigmine, Drug-Loaded Liposomes	Animal model	Intraperitoneal or intranasal injection of liposomes	Increased acetylcholine esterase inhibition and longer time of action
BDNF, Neurotrophic Factor-Loaded Liposomes	Animal model	Intravenous, -peritoneal, or -nasal injection of liposomes	Growth factor signaling
Donepezil, Microparticles	Animal model	Subcutaneous implantation of microparticles	Selective reversible inhibition of acetylcholinesterase
Neural stem cell, Biomaterials for Cell Transplantation	Animal model	Intracerebral injection of cell suspension and self-assembling peptide scaffold	Secretion of neurotrophic factors, differentiation to neuronal cells

### Nanoparticles

#### Polymeric Nanoparticles

Polymeric NPs, especially biodegradable NPs ranging in size from 10 to 1000 nm, are categorized as a major type of drug delivery system for the treatment of AD. This is due to their low toxicity, tunable degradation rate, and high drug loading capacity combined with their ability to cross the BBB and target the CNS (Wilson, 2009). Polymers include poly(lactic acid) (PLA), poly(glycolic acid) (PGA), poly(lactic-co-glycolic acid) (PLGA), poly(caprolactone) (PCL), chitosan, gelatin, and poly(butyl cyanoacrylate) (PBCA). Coating of these NPs with the surfactant polysorbate 80 enables them to cross the BBB by adsorption of apolipoprotein E from the blood, generating low-density lipoprotein mimics, which are taken up by cells of the BBB through receptor-mediated endocytosis (Wilson, 2009). Additionally, these types of NPs can have manipulated surface properties, such as a hydrophilic poly(ethylene glycol) (PEG) layer, which protects the loaded drug against recognition by the immune system or degradation by enzymes (Locatelli and Franchini, 2012). The above characteristics led to consider them as promising vehicles for the treatment of AD.

##### Growth Factor-Loaded Polymeric Nanoparticles

The interaction of growth factors (GFs) with their receptors on cell surfaces leads to activation of different signaling pathways. Each of these pathways is responsible for a different cellular behavior, such as survival, proliferation, and differentiation. Administration of GFs, such as insulin-like growth factor (IGF), basic fibroblast growth factor (bFGF), and nerve growth factor (NGF), which are normally present in the brain, shows beneficial effects on the pathophysiology of AD in animal models (Lauzon et al., 2015). However, delivery of GFs to the brain is highly challenging due to the BBB, sensitivity of GFs to enzymatic degradation, clearance, and denaturation in the blood and the brain (Di Stefano et al., 2011).

Kurakhmaeva et al. (2009) prepared NGF-loaded PBCA NPs of 250 nm coated with polysorbate 80. Intravenous administration of the particles in a mouse model of scopolamine-induced amnesia significantly improved memory function of the animals. Treatment with NGF-loaded PBCA NPs without polysorbate 80 coating also showed a positive effect, but less than with the surfactant.

Intranasal injection is a suitable administration route for NPs because of its large surface area, close distance to the brain, and high capillary density. By intranasal administration, particles are probably taken up by the olfactory epithelium and subsequently transported to the cerebrospinal fluid, thereby bypassing the BBB (Thorne et al., 2004). Zhang et al. (2014a) prepared PEG-PLGA NPs of 120 nm loaded with bFGF and coated with *Solanum tuberosum* lectin to target the nasal epithelium. Intranasal administration of the particles in a rat model of AD significantly improved spatial learning and memory capabilities of the animals. Intranasal NPs without lectin also showed positive effects, but less than with the lectin coating.

#### Nanoparticle Chelators

Abnormal metal ion homeostasis of the brain is also involved in the pathogenesis of AD and its characteristic Aβ neuropathology. Among other metal ions, enriched Fe, Zn, and Cu ions cause precipitation of Aβ and induce the formation of toxic Aβ oligomers (Adlard et al., 2008). This knowledge led to the development of a novel strategy to treat AD, based on metal-chelating compounds aiming to reverse or prevent Aβ aggregation. Moreover, these chelators can interfere with the role of redox metals in the generation of free radicals and oxidative stress, which results in neurodegeneration.

In an *in vitro* model using cultured human cortical neurons, administration of polystyrene NPs of 240 nm covalently conjugated with a deferiprone chelator resulted in decreased cytotoxicity of Aβ peptide by prevention of Aβ aggregation (Liu et al., 2009). This suggests that the use of metal-chelating compounds could be a powerful approach for the treatment or prevention of AD. However, the application of such agents is limited due to toxic side effects and low bioavailability due to low penetration potential of the chelators through the BBB. Both limitations can be improved by conjugation of the metal-chelating compounds to nanocarriers. It was demonstrated that the above polystyrene-chelator NPs were properly taken up by brain tissue *in vitro* without change in metal-chelating capacity (Liu et al., 2006).

In an *in vitro* study, the Cu (I) chelator d-penicillamine was covalently coupled by a disulfide bond to 70 nm lipid NPs made from hexadecanol and 1,2-dioleoyl-sn-glycero-3-phospho-ethanolamine-*N*-[3-(2-pyridyldithio)-propionate]. Metal-induced precipitation of Aβ was reversed by d-penicillamine released from the NPs by dithiothreitol (as a model for cellular glutathione), resulting in the formation of soluble deposits (Cui et al., 2005).

Another metal chelator is the quinoline derivative clioquinol (5-chloro-7-iodo-8-hydroxyquinoline) (CQ), which has affinity for Zn and Cu. Treatment of AD transgenic mice with CQ inhibited metal-induced Aβ aggregation (Cherny et al., 2001). A pilot phase 2 clinical trial showed the ability of orally administered CQ to slow down cognitive decline (Ritchie et al., 2003). In order to increase the bioavailability of CQ in the brain, the chelator was encapsulated in 40 nm PBCA NPs, which were coated with polysorbate 80. These CQ-NPs were found to efficiently cross the BBB in wild-type mice and thus have great potential for the treatment of AD (Roney et al., 2005).

#### Gold Nanoparticles

As described, destruction of Aβ fibrils and plaques is investigated as approach for the treatment of AD. This could also be achieved by the use of targeted gold NPs. Interaction of gold NPs with Aβ fibrils, and subsequent exposure to weak microwave fields, results in a local rise of temperature and dissolution of the fibrils. *In vivo* studies in mice demonstrated that treatment with gold NPs has the potential to slow down or stop the progression of AD, while these NPs cause no harmful effects in the brain (Lasagna-Reevesa et al., 2010). In a recent study, novel gold NPs conjugated with two compounds interfering with Aβ fibrils were used. These multifunctional 22 nm gold NPs were able to decrease the cytotoxicity of Aβ fibrils and Aβ-mediated peroxidase activity *in vitro* (Gao et al., 2015). Thus, these results indicate that inorganic multifunctional NPs have potential for the treatment of AD.

#### Liposomes

Liposomes are spherical vesicles that are composed of at least one relatively impermeable lipid bilayer surrounding an internal aqueous part. They usually consist of phospholipids such as phosphatidylcholine together with cholesterol. There is a considerable interest in liposomes due to their capacity of delivering hydrophilic and hydrophobic molecules, biocompatibility, and non-toxicity. Hydrophilic and hydrophobic compounds are, respectively, carried in the aqueous and lipid parts of the liposomes. Although liposomes are a type of foreign material, when introduced into the biological system they do not cause negative biological responses, and, in general, liposomes are non-immunogenic, non-carcinogenic, non-thrombogenic, and biodegradable (Gregoriadis, 2008). Liposomes as nanocarriers with a large transport capacity have been used for drug delivery to the brain. This is because liposomes can encapsulate different components, which are thereby properly protected against degradation by plasma enzymes and elimination by the reticuloendothelial system. Most importantly, liposomes are able to fuse with biological membranes, to be transported across cell membranes by endocytosis, and to penetrate through the BBB (Sinha et al., 2001). As discussed above for polymeric NPs, modification of the liposomal surface with PEG is a commonly used strategy to increase the half-life of liposomes in the circulation (Spuch and Navarro, 2011).

Gobbi et al. (2010) prepared liposomes with an average size of 145 nm that were targeted to Aβ by incorporation of phosphatidic acid and cardiolipin. The high affinity of these liposomes *in vitro* to Aβ oligomers, but not to monomers, suggests that they may be useful for the targeted delivery of diagnostic and therapeutic compounds in animal models of AD and clinical trials (Gobbi et al., 2010). Canovi et al. (2011) prepared PEGylated nanoliposomes functionalized with anti-Aβ monoclonal antibodies. The results showed significant binding of the liposomes to Aβ monomers and fibrils *in vitro*, with a twofold higher affinity for the fibrils. Moreover, the liposomes bound to Aβ deposits in post-mortem AD brain samples, indicating that this is a promising approach for the diagnosis and treatment of AD (Canovi et al., 2011). It should be noted that targeting of Aβ species in the blood may also be a valid strategy for lowering of Aβ levels in the brain, because blood and brain are in equilibrium through the BBB (Canovi et al., 2011).

Other potential applications of liposomes for the treatment of AD are based on their effects on cellular membranes. Addition of small (<200 nm) unilamellar phosphatidylcholine liposomes containing the omega-3 fatty acid, docosahexaenoic acid (DHA), to APP-overexpressing cells *in vitro*, resulted in an increase of cell membrane fluidity inducing the non-amyloidogenic processing of APP by α-secretase, leading to the formation of soluble APPα (sAPPα). Subsequently, sAPPα-containing cell supernatants were shown to inhibit the JNK stress signaling pathway and to activate the PI3K/Akt survival pathway in cultured neuronal cells, thereby preventing apoptotic cell death (Eckert et al., 2011). Thus, DHA-containing liposomes can potentially be used for the treatment or prevention of AD, by enhancing the non-amyloidogenic processing of APP.

##### Liposomal Encapsulation of Natural Components

Since there are no drugs to cure AD, current therapeutic strategies are based on inhibition of progress of the disease. Treatments are focused on targeting of metabolic dysfunction and the abnormal aggregation of tau proteins and Aβ peptides. (Citron, 2010). As discussed above, Aβ is initially released as monomer, which forms into oligomers, fibrils, and, eventually, a plaque. Although the oligomers are considered as the most toxic species, targeting of all Aβ forms is pursued for therapeutic and also diagnostic purposes (Spuch and Navarro, 2011).

For this approach, natural compounds could be used like curcumin, which is a substance from turmeric plants that has anti-inflammatory, antioxidant, and possibly anticancer properties, and might protect against AD. Liposomes with a size of 170 nm prepared from curcumin–phospholipid conjugates were shown to have a very high affinity to Aβ fibrils *in vitro*, with a much lower affinity to Aβ monomers (Mourtas et al., 2011). Curcumin-decorated liposomes only had a high affinity to Aβ fibrils, when the planar conformation of curcumin was maintained during preparation of the curcumin–phospholipid conjugates by means of click chemistry. It was concluded that these liposomes are promising vectors for the targeted delivery of therapeutic and diagnostic compounds in AD (Mourtas et al., 2011).

Quercetin, a flavonoid from fruits and vegetables, might also be useful for the treatment of AD. It has, among others, anticancer, anti-inflammatory, and antioxidant properties. As Aβ monomers are associated with oxidative stress-induced neurotoxicity, the antioxidant properties of quercetin may provide a protective or therapeutic effect in AD. Indeed, quercetin was shown to protect primary rat hippocampal neurons in culture from Aβ cytotoxicity, protein oxidation, lipid peroxidation, and apoptosis (Ansari et al., 2009). Although oral administration of quercetin in the mouse improved learning and memory ability, this was hampered by the low intestinal absorption, high metabolization, and rapid elimination of quercetin (Lu et al., 2006). To overcome this, quercetin was encapsulated in liposomes and administered *via* the nasal route. The results showed an antianxiety activity and a cognitive enhancing effect in the rat (Wattanathorn et al., 2007). Moreover, the same group showed that nasally administered quercetin-containing liposomes were able to inhibit the degeneration of hippocampal neurons in a rat model of AD, partly *via* reduction of oxidative stress (Phachonpai et al., 2010).

##### Liposomal Vaccines

Liposomes have also been used for the preparation of vaccines against AD. Muhs et al. (2007) incorporated a 15 amino acid sequence of Aβ peptide on the surface of liposomes, either by coupling of two fatty acid residues or two phospholipid/PEG spacers at both ends of the peptide. In the former case, the peptide adopted a β-sheet conformation, probably because of interactions of the peptide with the liposome surface. In the case of the PEG spacers, the peptide was present in a random coil conformation in the fluid phase surrounding the liposome. Intraperitoneal administrations in a transgenic mouse model of AD, only restored cognitive memory of the animals when a liposomal formulation containing the β-sheet form of the peptide was used. Moreover, in this case, a significant decrease in the amount of insoluble and soluble Aβ peptide in the brain of the mice was observed. These data show that AD is a “conformational” disease and that vaccination against β-sheet conformations of Aβ peptide has therapeutic potential.

##### Drug-Loaded Liposomes

Although the acetylcholinesterase inhibitor rivastigmine, which is FDA-approved for the treatment of AD, is well absorbed in the intestine, it has a half-life in blood of only 1.5 h. Therefore, the hydrophilic drug was encapsulated in phosphatidylcholine/cholesterol liposomes, and administered orally and intraperitoneally in mice. As compared to administration of the free drug, the use of liposomes increased both the acetylcholinesterase inhibitor activity in the brain and the time of action (Mutlu et al., 2011).

Rivastigmine was also incorporated in soya lecithin/cholesterol liposomes and administered in rats *via* the intranasal route. As compared to intranasally and orally administered free drug, intranasal liposomes resulted in a higher concentration and longer half-life of rivastigmine in the brain (Arumugam et al., 2008).

##### Neurotrophic Factor-Loaded Liposomes

Neurotrophic factors, such as brain-derived neurotrophic factor (BDNF), exert beneficial effects on neuronal survival and synaptic connectivity. Encapsulation in liposomes with appropriate surface properties, to increase the half-life of these factors *in vivo* and to facilitate targeting to the brain as described above, is considered as a viable strategy for the treatment of neurodegenerative diseases, such as AD (Spuch and Navarro, 2011).

### Microparticles

Donepezil is an FDA-approved acetylcholine esterase inhibitor with high specificity for acetylcholine esterase in the CNS. It significantly improves cognition and functioning in daily life of mild- to moderate-AD patients, without clinical changes in vital organ functions during long-term treatment (>98 weeks) (Rogers and Friedhoff, 1998). Currently, donepezil is available as a daily tablet. However, gastrointestinal side effects of this drug and difficulties related to daily intake of the drug in AD patients with impaired memory limit its effective application. This may be overcome by the development of a long-term, non-gastrointestinal delivery system. PLGA was used for the preparation of donepezil-loaded microparticles with a size of 75 μm (Zhang et al., 2007). Subcutaneous implantation of the particles in rats resulted in steady-state plasma levels of donepezil for 4 weeks, after which release of the drug rapidly declined due to exhaustion of the particles. The microparticles were also implanted in rats with permanent ligation of the common carotid arteries, which results in loss of neurons and decrease of learning and memory capabilities. It was shown that subcutaneous implantation of the donepezil-loaded PLGA particles significantly improved learning and memory deficits during 30 days, to the same extent as daily oral administration of the free drug. This indicates that the current treatment of AD with FDA-approved drugs may benefit from controlled release strategies (Zhang et al., 2007).

### Biomaterials for Cell Transplantation

Current treatments of AD are focused on alleviation of the symptoms, rather than trying to inhibit or reverse the progress of the disease. As AD is characterized by neuronal cell death, a viable approach for treatment would be to prevent the loss of functional neurons or to replace damaged neurons. With respect to the latter aim, the use of neural stem cells (NSC) has been proposed. It was shown that transplantation of NSC resulted in improved cognition and synaptic conductivity in animal models of AD (Blurton-Jones et al., 2009; Zhang et al., 2014b).

Although only cells can be injected into the brain, the use of a scaffold has advantages in terms of cell adhesion and guidance, as well as protection from the hostile environment consisting of Aβ oligomers and aggregates. In this respect, scaffolds prepared from the peptide RADA16 are very interesting. RADA16 is a peptide consisting of four repeats of the sequence arginine–alanine–aspartic acid–alanine. It has alternating hydrophilic and hydrophobic residues and self-assembles in the presence of physiological concentrations of monovalent salt ions, such as Na^+^ and K^+^, into nanofibrous structures. A nanofiber consists of two stacked β-sheets with a hydrophobic core, a hydrophilic surface, a width of 3–8 nm, and a height of around 1.5 nm (Holmes et al., 2000; Cormier et al., 2013). RADA16 scaffolds were shown to support neurite outgrowth and synapse formation *in vitro* (Holmes et al., 2000).

To increase the cell-supportive properties of these and other scaffolds, the peptide sequence YIGSR from the basement membrane protein laminin was used. When grafted on the surface of poly(ethylene-co-vinyl alcohol) membranes, YIGSR moieties increased the migration, differentiation, and synaptic activity of NSC *in vitro* (Li et al., 2013).

The C-terminus of the RADA16 peptide was extended with the YIGSR sequence, which did not interfere with self-assembly. As shown by Cui et al. (2016), these YIGSR-functionalized scaffolds promoted the neuronal differentiation of NSC and protected the cells against apoptosis by Aβ treatment *in vitro*, as compared to RADA16 control scaffolds. Moreover, hippocampal transplantation of NSC with RADA16-YIGSR scaffolds improved spatial learning and memory in a rat model of AD. This was accompanied by increased neuronal survival and differentiation, less apoptosis, recovery of synaptic function, and increased neurotrophin levels (Cui et al., 2016). These results indicate that the use of NSC together with biomaterials is a promising approach for the clinical treatment of AD.

### Time Course for Delivery of Therapeutic Agents and Selection of Biomaterials

In general, a degradable biomaterial is used for the delivery of therapeutic agents. Release of the active compound, which is dispersed in the biomaterial phase, is determined by degradation of the material, see the bFGF-loaded PLGA NPs in Section “Growth Factor-Loaded Polymeric Nanoparticles” (Zhang et al., 2014a). Thus, the degradation rate of the biomaterial must be fine-tuned to obtain the desired release profile of the therapeutic agent. This can be done by copolymerization. Both PLA and PGA have a relatively long degradation time (months), but PLGA degrades much faster (days–weeks). This is because PLA and PGA are crystalline materials, and PLGA is amorphous (Yang et al., 2001). The bFGF-loaded PLGA NPs (lactic acid:glycolic acid = 50:50) were daily administered in rats for 17 days *via* the nasal route, and peak concentrations of bFGF in the brain were reached 4 h after each administration (Zhang et al., 2014a). The degradation time of PLGA can be fine-tuned by varying the ratio of lactic acid:glycolic acid. The donepezil-loaded PLGA microparticles in Section “Microparticles” had a lactic acid:glycolic acid ratio of 75:25. These particles degraded completely within 30 days after subcutaneous implantation in the rat (Zhang et al., 2007).

Likewise, PBCA is a suitable polymer for the delivery of therapeutic agents, see the NGF-loaded PBCA NPs in Section “Growth Factor-Loaded Polymeric Nanoparticles” and the CQ-loaded NPs in Section “Nanoparticle Chelators.” PBCA shows relatively fast intracellular degradation (*T*_1/2_ = 3 days). If desired, the degradation time of this kind of particles can be increased by copolymerization of butyl cyanoacrylate with octyl cyanoacrylate (Sulheim et al., 2016). Both NGF- and CQ-loaded PBCA NPs were coated with polysorbate 80 and administered by single intravenous injection in mice. This resulted in maximum concentrations in the brain 1 h after administration (Roney et al., 2005; Kurakhmaeva et al., 2009).

Also, lipids are used for drug delivery, e.g., the solid lipid NPs in Section “Nanoparticle Chelators” conjugated with the Cu (I) chelator d-penicillamine. As shown *in vitro*, the chelator, which was covalently coupled to the particles *via* a disulfide bond, was only able to dissolve Aβ deposits after release from the particles. For this, dithiothreitol was used as a model for glutathione. Thus, although these particles are biodegradable, delivery of the active compound does not depend on degradation of the carrier (Cui et al., 2005).

Lipids are mostly used for controlled release in the form of liposomes, e.g., the unilamellar DHA-containing phosphatidylcholine liposomes in Section “Liposomes.” As shown *in vitro*, the cell membrane fluidity increased upon fusion with these liposomes (Eckert et al., 2011). The curcumin-decorated liposomes in Section “Liposomal Encapsulation of Natural Components” were prepared from phospholipids, cholesterol, and phospholipid–curcumin conjugates, and were shown to be stable *in vitro* for at least 24 h at 37°C in serum solutions (Mourtas et al., 2011). The quercetin-loaded liposomes in Section “Liposomal Encapsulation of Natural Components” were prepared from phosphatidylcholine and cholesterol and administered intranasally once daily for 4 weeks in the rat. Already after the first dose, a cognitive enhancing effect was measured, which further increased until the end of the study (Wattanathorn et al., 2007). The same type of liposomes was used to encapsulate rivastigmine (see Drug-Loaded Liposomes). As shown *in vitro*, the stability of these liposomes significantly increased by incorporation of sodium taurocholate in the phospholipid double layer. Intraperitoneal administration of these liposomes in mice resulted in a fivefold higher inhibition of acetylcholinesterase in the brain during the next 24 h, as compared to rivastigmine alone (Mutlu et al., 2011). For the liposomal vaccines (see Liposomal Vaccines), standard liposomes were used to present Aβ sequences with specific conformations to the immune system. Six intraperitoneal injections of the vaccine were given, with intervals of 2 weeks. The highest antibody titer (IgG) in blood was measured after the third inoculation in the case of Aβ peptide with a β-sheet conformation (Muhs et al., 2007). The liposomes can be expected to degrade within a few days after administration.

The scaffolds for transplantation of NSC in the brain were based on peptides that self-assemble into nanofibrous structures (see Biomaterials for Cell Transplantation). To prevent rapid degradation, the N- and C-termini of the peptides were acetylated and amidated, respectively. The nanofibers were stable for at least 2 weeks in cell culture. Learning and memory tests in a rat model of AD were carried out 4 weeks after injection of NSC and self-assembling peptides in the hippocampus (Cui et al., 2016). No data were provided about degradation of the scaffolds *in vivo*.

Sometimes, non-degradable polymers are used for drug delivery; see the polystyrene NPs conjugated with a deferiprone chelator in Section “Nanoparticle Chelators.” The chelator was covalently coupled to the particles, which were subsequently added to cultured neurons and brain tissue *in vitro* (Liu et al., 2006, 2009). Although the chelator was active after coupling, this raises questions about the fate of the non-degradable polystyrene NPs upon administration *in vivo*. The same holds for the gold NPs in Section “Gold Nanoparticles,” conjugated with compounds interfering with Aβ fibrils (Gao et al., 2015).

## Conclusion

Although many *in vitro* and preclinical studies have been published aiming to inhibit or reverse the progress of AD, current clinical treatments are still based on fighting the symptoms of the disease. Either approved or in the experimental phase, however, all treatment strategies may benefit from the use of biomaterials. These are mostly in the form of nano- or micro-sized particles that can protect the therapeutic payload after administration. Moreover, the biomaterial particles can facilitate transport of the therapeutic compounds across the BBB and target them to pathological sites in the brain. In addition to drugs, the active components can also be cells for regeneration of brain tissue. In this case, a biomaterial scaffold is used to guide and protect the cells. It is concluded that there is an increasingly important role for biomaterials in the treatment of AD.

## Author Contributions

Both DH and AP studied the current literature and wrote this review article.

## Conflict of Interest Statement

The authors declare that the research was conducted in the absence of any commercial or financial relationships that could be construed as a potential conflict of interest.
